# Increase in Protective Effect of *Panax vietnamensis* by Heat Processing on Cisplatin-Induced Kidney Cell Toxicity

**DOI:** 10.3390/molecules24244627

**Published:** 2019-12-17

**Authors:** Kim Long Vu-Huynh, Thi Hong Van Le, Huy Truong Nguyen, Hyung Min Kim, Ki Sung Kang, Jeong Hill Park, Minh Duc Nguyen

**Affiliations:** 1 Faculty of Pharmacy, Ton Duc Thang University, Ho Chi Minh City 70000, Vietnam; vuhuynhkimlong@tdtu.edu.vn (K.L.V.-H.); nguyentruonghuy@tdtu.edu.vn (H.T.N.); 2 Faculty of Pharmacy, University of Medicine and Pharmacy at Ho Chi Minh City, Ho Chi Minh City 70000, Vietnam; levan@uphcm.edu.vn; 3 College of Pharmacy, Seoul National University, Seoul 151-742, Korea; snuhmkim04@snu.ac.kr; 4 College of Korean Medicine, Gachon University, Seongnam-si 461-701, Korea; kkang@gachon.ac.kr

**Keywords:** *Panax vietnamensis*, ocotillol, ginsenoside, cisplatin, processed Vietnamese ginseng

## Abstract

Cisplatin is a platinum-based anticancer agent used for treating a wide range of solid cancers. One of the side effects of this drug is its severe nephrotoxicity, limiting the safe dose of cisplatin. Therefore, many natural products have been studied and applied to attenuate the toxicity of this compound. In this study, we found that steamed Vietnamese ginseng (*Panax vietnamensis*) could significantly reduce the kidney damage of cisplatin in an in vitro model using porcine proximal tubular LLC-PK1 kidney cells. From processed ginseng under optimized conditions (120 °C, 12 h), we isolated seven compounds (20(*R,S*)-ginsenoside Rh2, 20(*R,S*)-ginsenoside Rg3, ginsenoside Rk1, ginsenoside-Rg5, and ocotillol genin) that showed kidney-protective potential against cisplatin toxicity. By comparing the 50% recovery concentration (RC_50_), the *R* form of ginsenoside, Rh2 and Rg3, had RC_50_ values of 6.67 ± 0.42 µM and 8.39 ± 0.3 µM, respectively, while the *S* forms of ginsenoside, Rh2 and Rg3, and Rk1, had weaker protective effects, with RC_50_ ranging from 46.15 to 88.4 µM. G-Rg5 and ocotillol, the typical saponin of Vietnamese ginseng, had the highest RC_50_ (180.83 ± 33.27; 226.19 ± 66.16, respectively). Our results suggest that processed Vietnamese gingseng (PVG), as well as those compounds, has the potential to improve kidney damage due to cisplatin toxicity.

## 1. Introduction

Since its first discovery in 1978, cisplatin, a platinum-based alkylating compound approved by the United States Food and Drug Administration (USFDA), has become an antineoplastic agent for treating a wide range of solid cancers, such as metastatic testicular, ovarian tumors, and bladder cancer [1,2]. Cisplatin is currently available as a generic drug in the United States as a standard component of the treatment protocol of head and neck cancers, small cell and non-small cell lung cancer, cervical cancer, and others [3,4,5,6]. Although the precise cytotoxic mechanism of platinum compounds has not been fully elucidated, the interaction of cisplatin with DNA is observed when cells are exposed to cisplatin. The chloride ligands of cisplatin are replaced by a water molecule in an aqueous environment, forming a positively charged electrophile that reacts with nucleophilic sites to form DNA, RNA, and protein adducts. The binding of cisplatin and DNA leads to inter- and intrastrand crosslinks, resulting in the arresti of DNA synthesis and replication in rapidly proliferating cells. However, compared to other platinum-based anticancer drugs, cisplatin is considered the most toxic agent. Hence, these adverse effects, including toxicity on the cardiovascular, nervous, gastrointestinal as well as sensory systems, and myelosuppression, limit the dose of cisplatin used in cancer treatment [7]. Among those adverse effects, nephrotoxicity was classified as the most severe side effect of cisplatin, firstly reported in the 1971 [8]. Nephrotoxicity is clinically characterized by reduction of the glomerular filtration rate, increase of serum creatinine, and decrease of serum magnesium and potassium levels. Cisplatin may actively accumulate in renal parenchymal cells. Ctr1 and OCT2 are two membrane transporters that mediate the uptake of cisplatin into mammalian cells [9,10,11,12]. Animal studies indicate that cisplatin conjugates with glutathione to form an active metabolite that is transported into proximal tubule cells. Cisplatin induces both necrosis and apoptosis in kidney cells, leading to cell death [13]. Once in the cell, a highly reactive form of cisplatin interacts with thiol-containing molecules, thereby leading to the depletion or inactivation of glutathione. This, with the addition of mitochondrial dysfunction, may shift the cellular redox status and result in the accumulation of endogeneous reactive oxygen species (ROS) and oxidative stress. Cisplatin also induces myriad proinflammatory cytokines and chemokines, and causes an inflammatory response that contributes to the development of tissue damage and renal failure [14].

To overcome cisplatin-induced kidney demage, many protective strategies, ranging from inhibition of cisplatin metabolism, to anti-inflammating agent to reduce oxidative stress, have been used. Antioxidant reagents, such as *N*-acetyl cysteine, and vitamins C and E, have been shown to recover the kidney damage caused by cisplatin [15,16,17,18]. Natural products, such as tannin mixtures from green tea, quercetin, and gum arabic, also show nephroprotective effects against cisplatin [19,20,21]. There are many reports about the nephroprotective effect against cisplatin toxicity of processed Korean white ginseng (KWG) both in vitro and in vivo. Processing *Panax ginseng* at high temperatures and pressure generates not only less polar ginsenosides, such as G-Rk3, -Rh4, -Rg3, -Rg5, and -Rk1, but also non-ginsenoside compounds, such as the Maillard reaction product. These mentioned components can significantly reduce porcine proximal tubular kidney LLC-PK1 (Lilly Laboratories Cell-Porcine Kidney 1) cell damage by cisplatin through the regulation of inflammatory and apoptotic procedures [22,23].

Vietnamese ginseng (*Panax vietnamensis* Ha et Grushv., Araliaceae, VG) was first discovered in Ngọc Linh mountain, Quang Nam province, Vietnam in 1978 and since then has been considered the most southern and most recently found *Panax* species, with high commercial value on the herbal market. Chemical constituents of VG have been reported, with at least 52 saponins, including protopanaxadiol and protopanaxatriol-type saponins, such as G-Rb1, -Rd, -Rg1, and -Re, similar to *Panax ginseng*. More interestingly, VG contains a surprisingly high amount of ocotillol-type saponins, especially over 5% of majonoside R2 [24,25,26,27]. Pharmacological studies of VG have also been carried out to show the effect of VG on the central nervous system against stress, depression, and anxiety. Additionally, antitumor and hepatoprotective effects of VG have been reported [28,29]. However, the nephroprotective effect of VG and its main constituents, ocotillol-type saponins, is not well studied. An in vivo study on the protective effect of pseudo-ginsenoside F11 against cisplatin-induced kidney injury conducted by Wang et al. strongly suggested the potential nephroprotective effect of ocotillol-type saponins and their metabolite, ocotillol genin (OCT) [30].

In this study, we investigated the kidney protective effect of *Panax vietnamensis* after steaming. First, VGs were steamed at different time points. Then, the steamed ginsengs were analyzed by High-Performance Liquid Chromatography coupled with Quadrupole Time-of-Flight mass spectrometry detector (HPLC-QToF) and were assessed on kidney cell protective effects. The results showed that the changes in chemical profiles do affect the kidney cell protective effect, thus enabling the identification of an appropriate steaming time to increase the kidney cell protective effect of VG. In addition, we elucidated the active compounds from processed Vietnamese ginseng at selected steaming times and compared the kidney protective effect of these compounds. As a result, seven compounds possessing the highest kidney cell protective effects against cisplatin toxicity were identified, suggesting their potential not only in further mechanism studies but also in drug development. 

## 2. Results

### 2.1. Chemical Changes in Panax Vietnamensis by Steaming 

**Figure 1:**
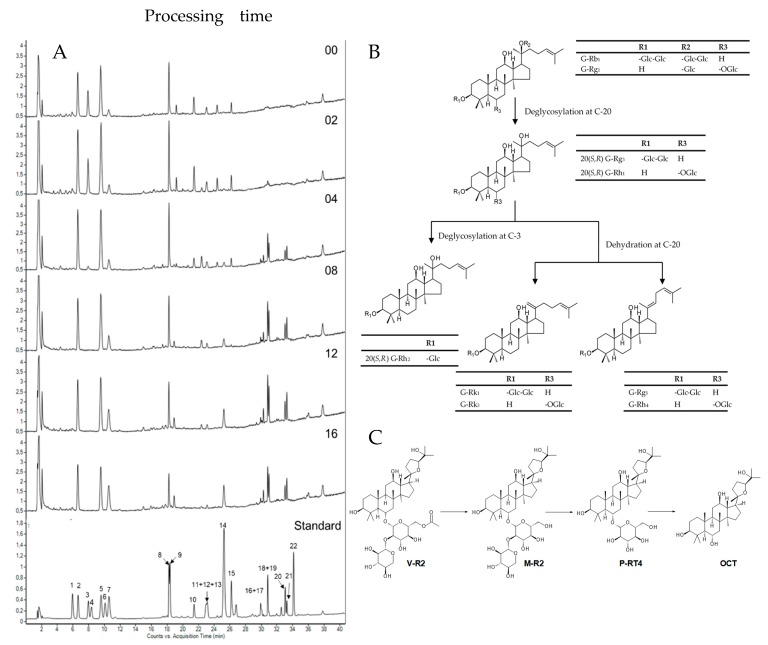
Chemical changes in *Panax vietnamensis* by the steaming process. (**A**) Representative HPLC-QToF chromatogram of the aqueous methanol extract of VG processed for 00, 02, 04, 08, 12, and 16 h, and the standard mixture. (**B**) Chemical modification of PPD- and PPT-type saponins during steaming. (**C**) Chemical modification of OCT-type saponins during steaming. Peak identities: 1, N-R1; 2, M-R1; 3, G-Rg1; 4, G-Re; 5, M-R2; 6, VR11; 7, p-RT4; 8, VR2; 9, VR1; 10, G-Rb1; 11 + 12 + 13, 20(R+S)-G-Rh1+Rc; 14, OCT genin; 15, G-Rd; 16 + 17, G-Rk3 + G-Rh4; 18 + 19, 20(R+S)-G-Rg3; 20; G-Rk1; 21, G-Rg5; 22, G-Rh2.

Observing the chromatograms at different steaming time points in Figure 1A, the contradictory trend of the peak intensity is noticeable. For instance, the first half of the chromatogram, from retention time of 4 to 22 min, shows a gradual decrease of the peak intensity from 0 to 12 h of steaming time, whereas the remaining half of the chromatogram, from the retention time of 24 to 38 min, clearly shows a gradual increase of the peak intensity from 0 to 12 h of steaming time. These differences could be explained, as during the heating process, the sugar moiety of these compounds was hydrolyzed, resulting in increases in the concentration of other less polar ginsenosides, such as 20(S,R)-G-Rg3, 20(S,R)-G-Rh1,-Rk3, -Rg5, -Rk1, and OCT. More information on these chemical changes can be found in Figure 1B,C. As a result, this reflects the changes in concentration due to the possible chemical transformation of compounds during the steaming process. 

### 2.2. Heating Increases the Kidney Cell Protective Effect of VG

**Figure 2:**
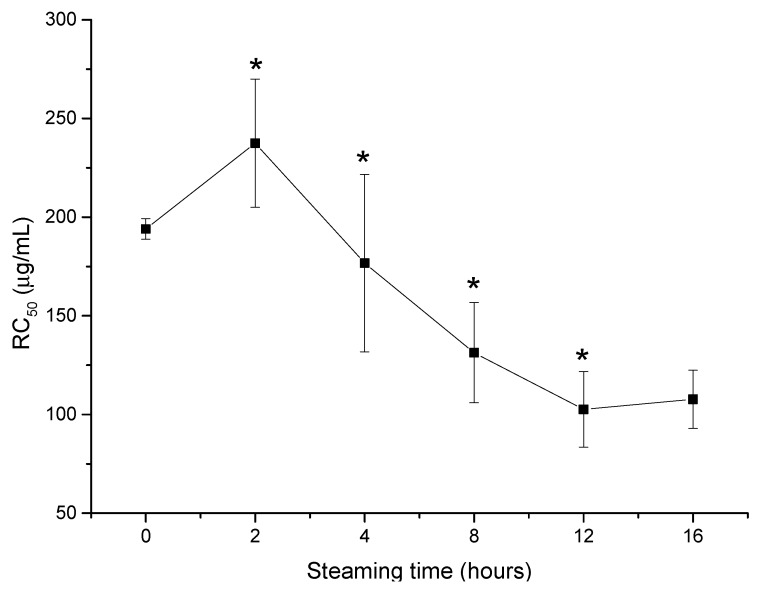
The decrease of the RC_50_ value of *P. vietnamensis* steamed at 120 °C for 0 to 16 h represents the increase in the kidney cell protective effect against cisplatin toxicity. Results are expressed as mean ± SD (n = 3), * *p* < 0.05, compared with the previous time of steaming (Student’s t-test). Concentrations are expressed as a weight of VG dry extract (µg) in 1 mL of the final medium.

To assess the change of the kidney cell protective effect of VG throughout the steaming procedure, LLC-PK1 cells were co-treated with the MeOH extract of PVG and steamed at 120 °C for 2 to 16 h and 20 µM cisplatin for 24 h. Cell viability was identified by water-soluble tetrazolium salt WST-8 reagent (Ez-Cytox) and measured at 450 nm. As shown in Figure 2, the kidney cell protective effect increased rapidly upon steaming and reached its maximum at 12 h, whereas raw VG extract showed a very mild effect. The increase of the steaming time to more than 12 h caused no significant change in the protective effect of VG extract. Therefore, steaming at 120 °C for 12 h was considered the optimal processing parameter to maximize the kidney cell protective effect of PVG against the toxicity of cisplatin. Additionally, we compared the recovery potential of PVG under the optimized condition with those of sun ginseng previously reported for kidney cell protective effects against cisplatin-induced renal injury [31]. PVG extract could recover 50% of cell loss due to cisplatin toxicity at a concentration of 105.7 ± 17.8 μg/mL, which is 1.5 times lower than that of Sun ginseng (SG, 159.3 ± 24.6 μg/mL). Moreover, at the maximum test concentration of 200 μg/mL, SG could recover 66.1% of the cells lost while that of PVG was 79.6 (data not shown). Finally, PVG extract at this condition was chosen for further bioactivity-guided fractionation and isolation.

### 2.3. Bioactivity-Guided Extraction

**Figure 3:**
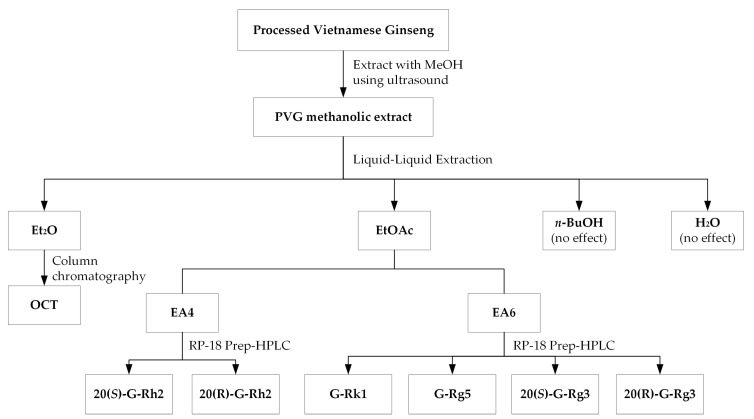
Scheme of the activity-guided fractionation and isolation of potential kidney cell protective compounds from PVG fractions against cisplatin toxicity on LLC-PK1 cells.

**Figure 4:**
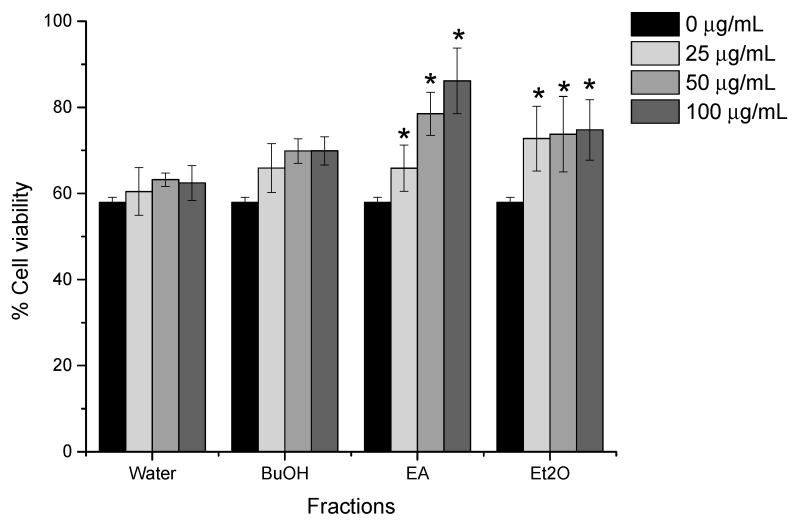
Kidney cell protective effect of PVG fractions against cisplatin toxicity on LLC-PK1 cells. Results are expressed as mean ± SD (n = 3), * *p* < 0.05, compared with the previous time of steaming (student’s t-test). Concentrations are expressed as a weight of VG dry extract (µg) in 1 mL of the final medium.

A scheme of the activity-guided fractionation and isolation of PVG is shown in Figure 3. Through a series of successive liquid-liquid extraction, the MeOH extract of PVG was separated into the diethyl ether (Et), ethyl acetate (EA), *n-*butanol, and aqueous fractions. Each fraction was introduced into the cell system as described above at three different concentrations (0, 25, 50, 100 µg/mL). The Et and EA fractions significantly increased cell viability (Figure 4). As a result, these two fractions were chosen for further isolation.

As shown in Figure 4, a portion of the Et_2_O extract was chromatographed on a silica gel column using a stepwise gradient elution of CHCl_3_/MeOH (100:0 → 5:1) and separated into seven fractions, namely fraction Et1–Et7. Among those, fraction Et5 exhibited potent protective activity in cells exposed to cisplatin. Through the separation and purification of fraction Et5 using a silica gel column eluted by CHCl_3_-MeOH (10:1), a bioactive compound was isolated and characterized as the ocotillol genin (OCT), the skeleton of ocotillol-type saponins that eliminates the sugar moiety.

Similarly, the EtOAc fraction was further separated into six fractions (EA1–EA6) using the mobile phase of CHCl_3_-MeOH-H_2_O (140:25:2.5 → 120:25:2.5 → 100:25:2.5 → 75:25:2.5). From the EA4 fraction, the bioactive compounds EA4.5 and EA 4.6 were isolated by RP-18 semi-preparative HPLC mobile phase acetonitrile (A) and water (B) (0–60 min, %A: 25%–95%), which were characterized as 20*(S)*-G-Rh_2_ and 20*(R)*-G-Rh_2_, successively. The EA6 fraction was suspended with MeOH, and 20*(R)-*G-Rg_3_ was consequently obtained in the form of a white precipitate powder. The remaining MeOH solution of EA6 was further isolated using RP-18 semi-preparative HPLC eluted by acetonitrile-water (53:47) to afford 20*(S)-*G-Rg3, -Rk1, and -Rg5, which showed the kidney protective effect on the LLC-PK1 system. The structures of all those bioactive compounds mentioned above were confirmed by comparing the retention time, high-resolution mass spectrum obtained from HPLC-QToF, and ^1^H, ^13^C-NMR data compared with published references [32,33,34]. 

### 2.4. Comparison of Protection Effects of Isolated Compounds

The 50% recovery concentrations (RC_50_) and the maximum recovery concentration of the isolated compounds (RC_max_) are shown in Table 1.

The data indicate that the *R* form of G-Rh2 and G-Rg3 showed the most potent protective effect, with an RC_50_ 8- to 10-fold lower than those of the *S* form. G-Rk1 exhibited a protective effect at 62.69 ± 17.3 µM, slightly higher than 20(*S)*-G-Rg3, its chemistry precursor. However, the RC_50_ of G-Rg5 was 180.83 ± 33.27, 3-fold higher than its isomer, G-Rk1. OCT, the aglycone of majonoside-R2, the characteristic saponin of VG, showed a 50% recovery concentration at 226.19 ± 66.16 µM. However, *N*-acetyl cysteine, used as the positive control, had an RC_50_ of 1543.6 ± 74.07, 7-fold higher than the RC_50_ of ocotillol genin. However, the protective ability against cell damage due to cisplatin toxicity of PPT, PPD, OCT-type saponins existing in raw VG (G-Rg1, -Rb1, -Rd, majonoside-R1, -R2, vina-ginsenoside-R1, -R2) could not be found in our LLC-PK1 model (data not shown)..

## 3. Discussion

Many types of processed Korean ginseng were previously reported to protect kidney cells from cisplatin toxicity. White ginseng could be steamed at 120 °C for 3 h or fermented with a microorganism, such as *Saccharomyces cerevisiae*, to obtain the less polar saponins [31,35]. In our study, the kidney cell protective effect increased gradually with the increase of time and reached a maximum at 12 h. Most of the less polar saponins possessing the kidney cell protective effect are protopanaxadiol-type saponins, including G-Rg3, -Rk1, -Rg5, -Rh2, and -Rh3 [22,35,36,37]. Only a few studies of the kidney protective effect of protopanaxatriol-type saponins (G-Rk3 and -Rh4) and ocotillol-type saponins (pseudoginsenoside F11) have been reported [30,31].

The steaming procedure was reported by Van et al. to significantly change the chemical constituents of raw Vietnamese ginseng, which increases less polar saponin, especially protopanaxadiol-type saponins, such as the *R* and *S* form of G-Rg3, G-Rh2, and ocotillol genin [38]. The highest kidney cell protective effect was observed after 12 h of steaming, which is parallel to the highest concentration of those mentioned less polar PPT-type saponins and ocotillol genin makers. On the other hand, the concentration that recovered 50% cisplatin-induced cell loss of PVG was 105.7 ± 17.8 μg/mL, about 1.5-fold lower compared with those of SG (159.3 ± 24.6 μg/mL). At the maximum test concetration of 200 μg/mL, PVG recovered 79% of cell loss, 1.2% higher than that of SG (66.1%). Furthermore, the RC_50_ of ocotillol genin was 226.19 µM, which was equivalent to 111.2 µg/mL, very similar to that of PVG (105 µg/mL). This difference in the RC_50_ of these two extracts could partly confirm the critical role of ocotillol genin present in PVG, which is absent in SG. Ocotillol-type saponins, including majonoside R2 and vina-ginsenoside R2, are considered the main constituents of Vietnamese ginseng. In our in vitro model, majonoside-R2 and vina-ginsenoside R2, the glycoside form of ocotillol genin, showed no activity. Due to their particular structure not having a heat-labile C-20 glycoside, these saponins were gradually degraded to ocotillol genin, which exhibited the kidney cell protective effect in the in vitro model. Moreover, pseudoginsenoside F11, a minor ocotillol-type saponin in *Panax ginseng*, was reported to have a protective effect against cisplatin-induced acute renal failure in an in vivo model [30]. These findings could be explained by a study by Jeong et al., which described the metabolism of these saponins to ocotillol genin by gut microbiota in a human faecal suspension [39]. This suggests that the kidney cell protective effect of ocotillol-type saponins mainly comes from the genin form, which is produced by the digestive procedure in human intestines. Besides, this study also reported ocotillol genin as a potential anti-inflammatory agent in an lipopolysaccharide-stimulated peritoneal macrophages model. Therefore, it could be hypothesized that ocotillol genin could ameliorate the kidney injury status by suppressing the inflammatory response induced by cisplatin. However, more data from further mechanism experiments are required to confirm this hypothesis.

VG shares similar chemical constituents with PG regarding protopanaxadiol- and protopanaxatriol-type saponins. Therefore, from VG, some PPD-type less polar ginsenosides, such as G-Rg3, -Rk1, -Rg5, and -Rh2, displayed kidney cell protective effects, which is consistent with previous reports [22,37]. Especially, G-Rh2, a rare PPD-type saponin, exhibited the ability to recover cisplatin-induced kidney cell loss at a surprisingly low dose. G-Rh2 was also previously reported to hav a cardioprotective effect against the toxicity of doxorubicin as well as enhance the antitumor activity of cyclophosphamide and also decrease the genotoxic of this reagent [40,41]. The protective effect may be due to the antioxidant characteristic of PPD-type saponins, which could scavenge the free radical induced by chemotherapy reagents, such as cisplatin, cyclophosphamide, or doxorubicin. Also, the less polar structure of G-Rh2 compared to G-Rg3, G-Rk1, or G-Rg5 could facilitate the diffusion of the molecule to the cells, which could explain its high efficiency at a lower dose than the other PPD-type saponins.

There are many reports about the differences in bioactivity of the isomers of a single compound, for example, G-Rg3, -Rh2, -Rk1, and -Rg5 are isomeric compounds. However, previous studies report their nephroprotective effect as a mixture of the two isomers or only the *S* form of those compounds [22,35,40]. In this study, we investigated the kidney cell protective effect of the isomers of these compounds. Interestingly, the *R* form of G-Rg3 and -Rh2 was 8- to 10-fold more active than their *S* form. Also, G-Rk1 was three times more active than its isomer, G-Rg5. 20(*R*)-G-Rg3 was reported to possess free radical scavenging as well as angio-suppressive effects [42,43]. Wei et al. studied the antioxidant effect of the *S* and *R* form of G-Rg3 on oxidative stress induced by cyclophosphamide in mice [44]. The results determined that the *R* form possesses a significantly higher antioxidant effect than *S* form, which is consistent with our data. These results suggest that there is a structure–activity relation in the stereotype or the position of double bonds. 

## 4. Materials and Methods 

### 4.1. Materials

Vietnamese ginseng (VG) was collected at Tra Linh Farm, Quang Nam Province in 2016. A voucher specimen was deposited at the herbarium of College of Pharmacy, Seoul National University, Seoul, Korea. In total, 1.5 kg of fresh VG roots were then dried at 40 to 60 °C and subsequently ground and sieved to obtain a powder with particles lower than 425 µm.

Sun ginseng was generously provided by Ginseng Science Inc. (Seoul, Korea). Sun ginseng is heat-processed ginseng at high temperatures and pressures, resulting in G-Rg3, -Rk1, and -Rg5 being its main ginsenoside components [31].

### 4.2. Methods

#### 4.2.1. Preparation of Processed Vietnamese Ginseng at Different Times

A 100-mg portion of the VG powder sample was put into a stainless steel vessel with 1 mL of distilled water. The vessel was closed tightly and heated in an oven for 2, 4, 8, 12, or 16 h at 120 °C (n = 3). After heating, all the samples were lyophilized to yield a dried powder and then extracted six times, each time with 3 mL of methanol (MeOH), by ultrasonication at room temperature for one hour. The extracts were centrifuged at 3000 rpm for 5 min, combined, and dried under reduced pressure to obtaine dried processed Vietnamese ginseng (PVG) extract. Dry PVG extracts were dissolved in DMSO to make stocks of 100 mg/mL and further used for cell treatment.

#### 4.2.2. Processed Vietnamese Ginseng Extraction and Isolation

VG dried powder (50 g) was suspended in 200 mL of distilled water and steamed at 120 °C for 12 h to obtain PVG powder, which was then lyophilized and extracted six times by sonication with 200 mL of MeOH. The organic solvent of the methanolic extract of PVG was removed under reduced pressure, and a portion of the residue was suspended with water and extracted with diethyl ether (Et_2_O), ethyl acetate (EtOAc), and water-saturated *n*-butyl alcohol (BuOH), successively. Each organic or aqueous extract was evaporated under reduced pressure and stored in a −20 °C refrigerator. Each fraction was then subjected to column chromatography using silica gel (230–400 mesh, Merck, Darmstadt, Germany). The pure compounds were isolated by semi-preparative chromatography using a Gemini C18 column (250 × 10 mm, 5 µM, Phenomenex, Torrance, CA, USA), on a Gilson chromatograph equipped with a Gilson pump 321, a Gilson UV/Vis-155 detector set at 210 nm, and a fraction collector FC204 (Gilson, Middleton, WI, USA).

#### 4.2.3. Liquid Chromatography–QToF Mass Spectrometry Analysis

The instrumental analysis was performed with the Agilent 1260 HPLC system (Santa Clara, CA, USA) using a Kinetex C18 column (50 × 4.6 mm. i.d., 2.6 µm, Phenomenex Torrance, CA, USA) at 25 °C. The binary gradient elution system consisted of 0.1% formic acid in water (A) and 0.1% formic acid in acetonitrile (B). The separation was achieved using the following protocol: 0–10 min (22% B), 15–20 min (31% B), 25 min (40% B), 30 min (60% B), 45–50 min (95% B), and 51–60 min (22% B). The flow rate was kept at 0.3 mL/min, and the sample injection volume was 2.0 µL. The metabolite profiling of PVG was performed on Agilent 6530 QTOF-MS equipped with an electrospray ion source with an Agilent Jet Stream Technology system (Agilent, CA). The source parameters were as follows: Capillary voltage of 3.5 kV, nebulizer pressure of 45 psi, dry gas flow of 5 L/min, and dry gas temperature of 300 °C. The ion transfer and collision stages were set as follows: Nozzle voltage of 500 V, and fragmentor of 250 V at positive mode. High purity nitrogen was used as a nebulizer gas, dry gas, and collision gas. Then, 1.5 µL of PVG stocks were diluted with 1500 µL of HPLC-grade MeOH, filtered with a 0.2-µm PTFE filter (Ottawa, Tokyo, Japan) to obtain 100 ppm samples. A standard solution containing 16 reference ginsenosides was prepared in pure MeOH. Then, 2 µL of each sample were injected into the HPLC-QToF system.

#### 4.2.4. Nuclear Magnetic Resonance (NMR) Spectroscopy

NMR spectra were obtained using a NMR-spectrometer (Bruker, Billerica, MA, USA), samples were dissolved in pyridine-*d*5. MS spectra were obtained using Agilent-QToF mass spectrometry under positive and negative mode with 250 voltage. The structure of those isolated compound was verified by comparing them with data from published references [32,33,34].

#### 4.2.5. Cell Culture and Cells Viability Assay

LLC-PK1 cells (ATCC, Manassas, VA, USA) were cultured in a DMEM-Glutamax-I medium supplemented with 10% fetal bovine serum, and antibiotics (100 units/mL of penicillin G and 100 µg/mL streptomycin) (Gibco, Grand Island, NY, USA). Cells were maintained in a humidified 5% CO_2_ incubator at 37 °C. When the cells were ~80% confluent, they were seeded in 96-well culture plates at 1 × 10^4^ cells per well and incubated for 24 h for adhesion. Then, cells were treated with the determined concentrations of extracts, fractions, or pure compounds. After incubation for 2 h, 20 µM cisplatin was added to each well and further incubated for 24 h. The final concentration of cisplatin was 20 µM, and that of DMSO was 0.2%. After incubation, 10 µL of WST-8 reagent (Ez-Cytox, DOGEN Bio Co., Ltd., Seoul, Korea) was added to each well and incubated for 2 h. Cell viability was measured by absorbance at 450 nm using a microplate reader (SpectraMax 190, MolecularDevice, San Jose, CA, USA). Percent of recovery was calculated by recovery(%) = (%SC – %C)/(100 – %C) × 100, where %SC and %C is the percentage of cell viability of cisplatin-sample co-treatment groups and cisplatin only groups, respectively, in comparison with those of th vehicle. The 50% recovery concentration (RC_50_) was calculated based on the linear regression of the plot of means value (n = 3) of the percent recovery of the six concentrations of the corresponding compounds. The results are expressed as mean ± SD. The maximum recovery concentration (RC_max_) was determined by the concentration at which the cell viability was the highest.

## 5. Conclusions

In conclusion, we demonstrated the optimized condition to process Vietnamese ginseng, which archived the highest kidney cell protective effect against cisplatin toxicity in vitro. Additionally, from processed Vietnamese ginseng, we isolated six PPD-type saponins and ocotillol genin that displayed the potential to protect the kidney from cisplatin-induced toxicity, with RC_50_ valus ranging from 6 to 226 μM.We also demonstrated that the *R* form of PPD-type saponins has a stronger protective effect than the *S* form. Further study should be carried out to examine these compounds, especially ocotillol genin, in in vivo experiments to investigate the protection of the kidney as a whole body system and to elucidate the mechanism of the protective effect of these compounds.

## Figures and Tables

**Table 1:** RC_50_ value of isolated compounds.

No.	Compounds	RC_50_ (µM) ± SD	RC_max_ (µM)/Recovery Rate (%)
1.	20(*R*)-Ginsenoside-Rh2	6.67 ± 0.42	10/73.9
2.	20(*R*)-Ginsenoside-Rg3	8.39 ± 0.3	25/69.9
3.	20(*S*)-Ginsenoside-Rh2	46.15 ± 9.66	50/75.7
4.	Ginsenoside Rk1	62.69 ± 17.3	50/40.6
5.	20(*S*)-Ginsenoside-Rg3	88.4 ± 54.62	200/59.3
6.	Ginsenoside Rg5	180.83 ± 33.27	200/43.5
7.	Ocotillol genin	226.19 ± 66.16	200/43.9
8.	*N*-acetyl cysteine	1543.6 ± 74.07	4000/67.6
